# Helicobacter pylori-Negative Gastric Mucosa-Associated Lymphoid Tissue Lymphoma Presenting as Massive Gastrointestinal Bleed

**DOI:** 10.7759/cureus.29125

**Published:** 2022-09-13

**Authors:** Syed Hamaad Rahman, Ali Waqar Chaudhry, Sadaf Raoof, Nihal Khan, Abu H Khan

**Affiliations:** 1 Internal Medicine, Methodist Health System, Dallas, USA; 2 Medicine, FMH College of Medicine and Dentistry, Lahore, PAK; 3 Internal Medicine, AdventHealth Orlando, Orlando, USA; 4 Internal Medicine, Allama Iqbal Medical College, Lahore, PAK; 5 Gastroenterology and Hepatology, AdventHealth Orlando, Orlando, USA

**Keywords:** primary gastric lymphoma, hpylori gastritis, massive gastrointestinal bleeding, helicobactor pylori, gastric maltoma

## Abstract

This case reports a patient that represents the minority of patients with gastric mucosa-associated lymphoid tissue (MALT) lymphoma who do not have underlying Helicobacter pylori gastritis. Gastric MALT lymphoma is a type of primary gastric lymphoma (PGL), which are extremely rare gastric malignancies characterized by proliferation of B-cells and infiltration of lymphoid tissue leading to destruction of gastric glands. Development of gastric MALT lymphoma is associated with H. pylori gastritis. Patients typically present with a wide range of symptoms including but not limited to epigastric pain, weight loss, gastrointestinal bleeding and gastric wall perforation. Gastric MALT lymphoma presenting as a massive gastrointestinal bleed is quite rare and only a few cases have been documented. Our case demonstrates that it is important to recognize that acute presentations of this disease may also occur.

## Introduction

Primary gastric lymphomas (PGL) are exceedingly rare, making up approximately 3% of gastric neoplasms and 10% of all lymphomas [1]. PGLs are divided into mucosa-associated lymphoid tissue (MALT) lymphomas and diffuse large B-cell lymphomas (DLBCL) [2]. MALT lymphomas account for 40% of PGLs. The incidence of developing PGL is two to three times higher in males than females and the highest incidence of developing MALT lymphoma occurs between the ages of 50 and 60 years [3]. MALT lymphoma is considered a low-grade neoplasia with infiltration of B-cells in lymphoid tissues, resulting in destruction of gastric glands [4]. There is a strong association between MALT lymphoma and Helicobacter pylori. Patients with gastritis secondary to H. pylori infection are at an increased risk of developing gastric MALT lymphomas [5]. Because of its nonspecific clinical presentation, gastric MALT lymphoma may be challenging to diagnose. Patients can present with dyspepsia, nausea, vomiting, abdominal pain, weight loss, gastrointestinal bleeding, and rarely gastric wall perforation. In some cases, patients may experience typical B-cell lymphoma symptoms such as night sweats [3]. The gold standard of diagnosis is endoscopic biopsy [6]. 

In this paper we present a case of H. pylori-negative gastric MALT lymphoma presenting as an overt gastrointestinal bleed. 

## Case presentation

**Figure 1:**
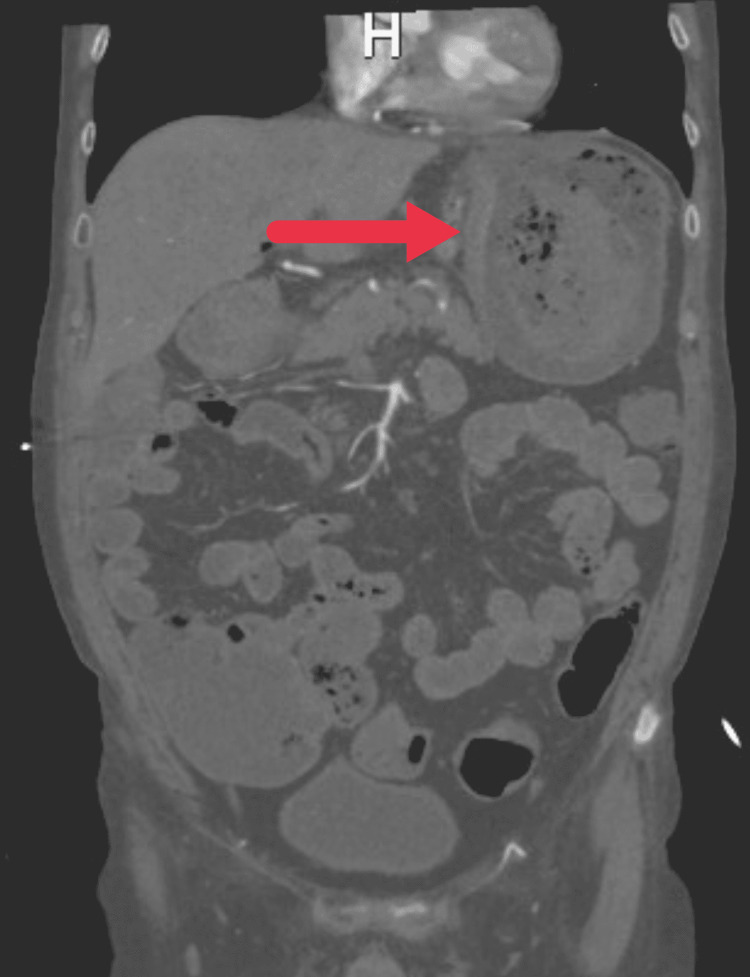
Focal wall thickening (red arrow) along the proximal lesser curvature of stomach.

A 75-year-old male with a past medical history of hypothyroidism, hypertension, and hyperlipidemia presented to the emergency department after having a syncopal episode in his bathroom. Prior to arriving at the hospital, he was complaining of low back pain, dark bloody bowel movements, and weakness. Emergency medical services were called and noted that when they arrived at the scene the patient was semi-conscious, pale, and hypotensive with a blood pressure of 58/29. Upon arrival at the hospital, intravenous fluids and vasopressors were administered to the patient. Laboratory tests were notable for anemia with a hemoglobin and hematocrit of 8.6 and 27.5 respectively, hyperkalemia with a potassium of 5.8, elevated lactic acid of 3, and mild elevation of alanine aminotransferase (ALT) and aspartate aminotransferase (AST) of 67 and 109 respectively. All other laboratory tests were within normal limits, including white blood cells and platelets. The patient had another large bloody bowel movement shortly after arrival and was given 1 unit of packed red blood cells (PRBCs). A CT scan of the abdomen and pelvis with contrast was obtained which was notable for a deep gastric ulcer along the proximal lesser curvature of the stomach (Figure 1), associated with adjacent gastric hepatic ligament lymphadenopathy.

**Figure 2:**
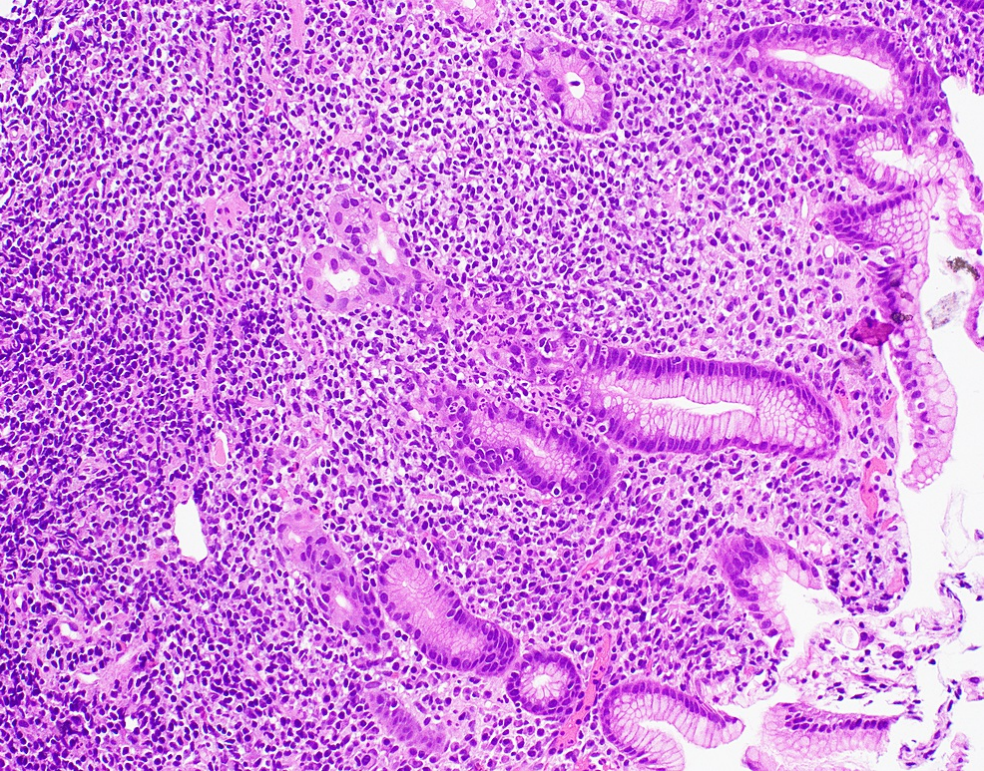
Hematoxylin and eosin stain 20x showing atypical lymphocytic infiltrate.

**Figure 3:**
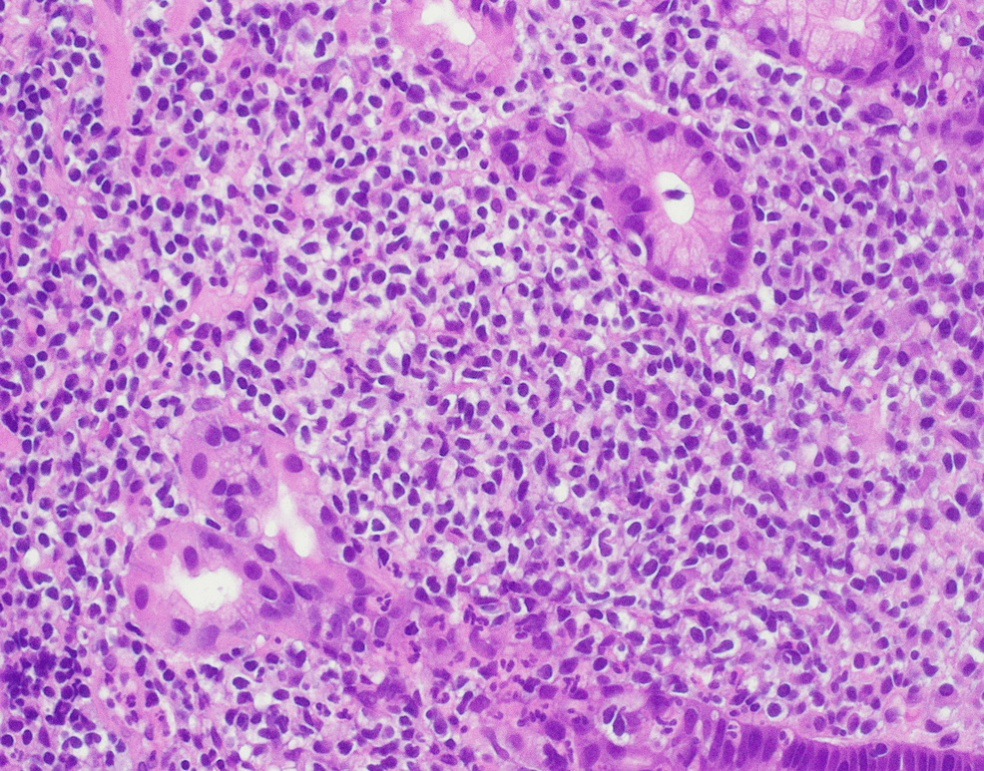
Hematoxylin and eosin stain 40x showing atypical infiltrates.

**Figure 4:**
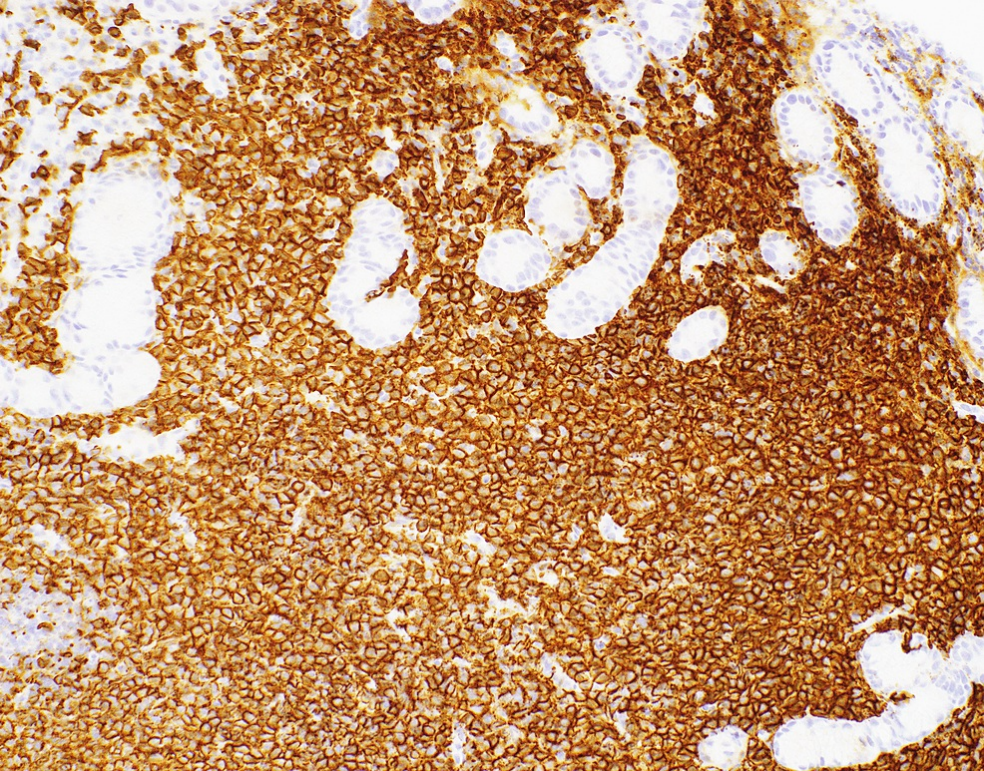
CD20 stain 20x showing increased B lymphocytic infiltrate.

**Figure 5:**
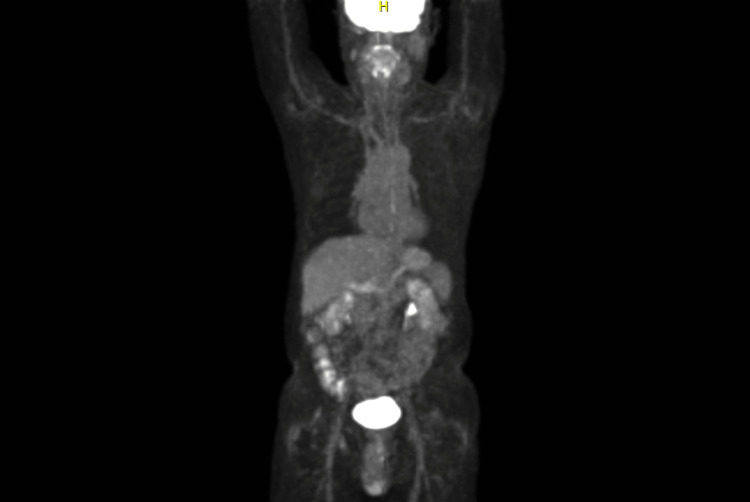
PET scan Positron emission tomography (PET) scan did not show any signs of metabolic activity in the stomach or at distant sites. Given that histopathological analysis of the biopsy was indicative of low-grade gastric mucosa-associated lymphoid tissue (MALT) lymphoma, it would be unlikely for the primary gastric lesion to show up on PET scan. Studies show that PET/CT for MALT lymphoma of the stomach detects active disease in the stomach only about 50% of the time.

An esophagogastroduodenoscopy (EGD) was performed which revealed multiple non-bleeding gastric ulcers with visible vessels, which were treated with cautery. Biopsies of the ulcers were taken for further evaluation. Histopathological analysis of the biopsy samples identified atypical B-lymphocytic infiltrate (Figures 2, 3). CD20 and PAX5 immunohistochemistry staining of the sample highlighted increased number of B-lymphocytes which expressed CD43 (Figure 4). These B-lymphocytes were positive for BCL2 and negative for CD10, BCL6, and cyclin D1. Immunoglobulin heavy chain gene (IGH) rearrangement testing was positive. Fluorescence in situ hybridization (FISH) testing revealed t(11;18) translocation, characteristic of MALT lymphoma. Biopsy samples did not detect H. pylori. Stool antigen test for H. pylori was negative. Urea breath test for H. pylori was done twice and both times resulted negatively. A positron emission tomography (PET) scan did not demonstrate any metastasis to distant organs (Figure 5). Bone marrow biopsies were also negative. 

The patient's disease was staged as IE and surveillance strategy vs radiation therapy was discussed. Chemotherapy was not discussed as an option for treatment given the early staging and indolent nature of the disease based on pathology and imaging. Immunotherapy with rituximab was considered, however this approach was not selected given his localized disease that could be treated in a single radiation field. Furthermore, given our patient's advanced age, it was decided to avoid rituximab due to its immunosuppressant effects that could increase his risk of complications from infections. Antibiotic eradication therapy was discussed with the patient, including his t(11;18) translocation and the lower likelihood of a complete response to antibiotic therapy. It was explained to the patient that only about 30% of patients in his situation would be expected to maintain a complete response at five years. The patient elected to proceed with conservative approach of antibiotic eradication triple therapy given his advanced age and desire to minimize adverse reactions from other treatment modalities discussed. The patient was instructed to follow up with hematology and oncology to discuss further treatment options and repeat EGD will be performed after two months to assess the efficacy of eradication therapy. If eradication therapy was not successful, radiation therapy will be considered. 

## Discussion

Gastric MALT lymphoma is a rare disease characterized by proliferation of B-cells and infiltration of lymphoid tissue leading to destruction of gastric glands. Patients typically present with a wide range of symptoms including but not limited to nausea, vomiting, epigastric pain, weight loss, gastrointestinal bleeding, gastric wall perforation, and sometimes B-symptoms such as night sweats [4]. The association between gastric MALT lymphoma and H. pylori has been well documented. Approximately 90% of patients with gastric MALT lymphoma are H. pylori positive. H. pylori plays a crucial role in the pathogenesis of the disease, specifically in stimulating the proliferation of B-cells. In patients with H. pylori infection, B-cells undergo genetic changes including chromosomal translocations causing malignant transformation and dysregulation of inflammatory and apoptotic agents such as nuclear factor kappa B (NF-κB) and Bcl2, respectively [5]. Development of the disease is influenced by genetic as well as environmental risk factors. A number of alleles have been found to be associated with incidence of gastric MALT lymphoma. Obesity, metabolic syndrome, hyperglycemia, insulin resistance, and high dietary salt intake are environmental factors associated with development of the disease. Infectious agents such as hepatitis B virus, human immunodeficiency virus (HIV), Epstein-Barr virus and human T- cell lymphotropic virus type 1 were also found to be associated [4]. 

Endoscopy is a routine diagnostic procedure in cases of gastric MALT lymphoma and biopsy is the gold standard of diagnosis [6]. Endoscopic findings are nonspecific and can include gastric erosions/ulcerations, thickened gastric folds, petechial hemorrhages, and nodularities. Magnifying endoscopy can also be utilized to look for destroyed gastric pits. Endoscopic ultrasonography (EUS) is also used to assess the extent of gastric wall involvement [7]. Biopsy samples are then tested for H. pylori via urease test and histological analysis. The most commonly involved site is the antrum [4]. Once the diagnosis has been established, staging of the disease is critical. Imaging including CT scans, EUS, and PET scans play an important role. Studies show that PET scans for gastric MALT lymphoma have a detection rate of only about 50% for active lesions in the stomach. There are multiple staging criteria that have been developed, the most commonly used one being the Ann Arbor Classification of Gastric Lymphoma (Table 1). Advanced stages are found in approximately 10% of diagnosed patients and require anti-neoplastic treatment [4].

**Table 1:** Ann Arbor Classification of Gastric Lymphoma (Musshoff Modification) The most commonly used staging criteria is the Ann Arbor Classification of Gastric Lymphoma [4].

Stage	Description
IE	restricted to the stomach
IE1	limited to mucosa and submucosa
IE2	spread to submucosa
IIE	spread to the lymph nodes
IIE1	spread to the perigastric lymph nodes
IIE2	spread to the sub-diaphragmatic lymph nodes
IIIE	spread to the lymph nodes on both sides of the diaphragm
IVE	spread to the extra-gastrointestinal tissues or organs

Prognosis is determined based on the MALT lymphoma prognostic index (MALT-IPI) which claims that patients who are > 70 years of age, have elevated lactate dehydrogenase (LDH), and Ann Arbor Stage III or IV are the most important factors for indicating a poor prognosis. The t(11;18) translocation has also been associated with poor prognosis in patients due to antibiotic resistance [8]. Therapeutic regimens targeting H. pylori have been shown to improve survival rates [4]. 

Treatment of the disease is determined by the stage of the disease. Treatment in patients who are in the early stages of the disease and are found to be H. pylori positive is focused on eradicating the infection. The first-line regimen for these patients consists of triple therapy (proton pump inhibitor, clarithromycin, and amoxicillin/metronidazole) for seven to 14 days, as recommended by the American College of Gastroenterology [9]. There are no clear guidelines for the use of antibiotic therapy in patients found to be H. pylori negative, however recent studies indicate that H. pylori eradication may be useful as a treatment for gastric MALT lymphoma even in patients who are H. pylori negative [3,10]. One systematic review with a pooled analysis of 11 studies with 110 H. pylori-negative patients showed that complete lymphoma regression was achieved with eradication therapy in approximately 15.5% of patients [11]. In a meta-analysis by Jung et al. which included 25 studies, the overall pooled rate of complete remission in H. pylori-negative gastric MALT lymphoma patients treated with eradication therapy was 29.3% [12]. Another meta-analysis by Xie et al. including 15 studies described a pooled response rate of 57% to eradication therapy for H. pylori-negative gastric MALT lymphoma patients from Western countries [13]. The mechanism behind these findings is unclear, however a few hypotheses have been proposed. One theory is that these patients were in fact H. pylori positive despite having negative testing (false negative). Others have suggested that there may be another underlying infection by a different infectious agent causing the same malignant transformation that H. pylori would, and treatment with antibiotic therapy eradicates that infectious agent. Some studies have emphasized the impact of clarithromycin on the patient’s immune system as a possible explanation for these findings. It is important to note that utilization of H. pylori eradication therapy is not currently standard of care for H. pylori-negative gastric MALT lymphoma patients. The current standard of care for these patients is based on staging of the disease and includes involved site radiation therapy (ISRT) and rituximab, per the National Comprehensive Cancer Network (NCCN) guidelines. Further randomized-prospective studies need to be developed to further explain the role of eradication therapy in patients who are H. pylori negative. In any case, these findings are significant because the alternative treatments to eradication therapy include radiotherapy, chemotherapy, and monoclonal antibody treatment, which are all more expensive and come with more risks and side effects compared to eradication therapy [3,10,11].

The majority of patients are cured by eradication therapy alone. Patients who are in more advanced stages of the disease or are not fully cured by eradication therapy alone can be treated with chemotherapy/immunotherapy, radiation, and sometimes even surgery. Chemotherapy and monoclonal antibody treatment (e.g. rituximab) remain the major mainstays of treatment for gastric MALT lymphoma. Rituximab combined with chemotherapeutic agents has shown to have the most superior results. One large, randomized study (IELSG-19) comparing chemotherapy alone versus chemotherapy plus rituximab showed higher complete remission rates (78% vs 65%) and significantly better 5-year event-free-survival rates (68% vs 50%) for patients who received combination therapy. Radiation therapy has also played a useful role in treatment, with remission rates of 93 to 100%. Surgery is generally reserved for cases with massive gastrointestinal bleeding that cannot be resolved on endoscopy, gastric wall perforation, and other damaging anatomic deformities caused by the extent of infiltration into the gastric wall [3,4]. Endoscopic follow-up examination with biopsy should occur within three to six months following eradication therapy and is an essential step for establishing remission. The Groupe d’ Etude des Lymphomes de l’ Adulte (GELA) developed a classification system for determining remission in such patients in 2012 (Table 2) [14].

**Table 2:** GELA classification system The Groupe d’ Etude des Lymphomes de l’ Adulte (GELA) developed a classification system for determining remission on endoscopic follow up examination with biopsy within three to sixmonths following eradication therapy in 2012 [12].

Classification	Lymphoid infiltrate
Complete Remission (CR)	Absent or scattered plasma cells and small lymphoid cells in LP.
Probable minimal residual disease (pMRD)	Aggregates of lymphoid cells or lymphoid nodules in LP/MM and/or SM.
Responding residual disease (rRD)	Dense, diffuse, or nodular extending around glands in LP.
No change (NC)	Dense, diffuse, or nodular

Our patient represents the minority of patients who do not have underlying H. pylori gastritis and are diagnosed with gastric MALT lymphoma. This case highlights the possibility of utilizing H. pylori therapy in an H. pylori-negative patient prior to initiating more aggressive therapeutic modalities. Furthermore, this case is interesting in that our patient’s initial presentation leading to diagnosis was a massive gastrointestinal bleed that required aggressive resuscitation. PET scan is regularly utilized in the staging process of this disease, however studies show that PET/CT for gastric MALT lymphoma detects active disease in the stomach only about 50% of the time [15]. This explains why the PET scan for our patient did not show metabolic activity at the site of the primary gastric lesion. This presentation of gastric MALT lymphoma is quite rare and only a few cases have been documented. While many patients who are diagnosed with gastric MALT lymphomas present with more chronic symptoms such as gastrointestinal upset or abdominal pain, our case demonstrates that it is important to recognize that acute presentations of this disease may also occur (Table 3). 

**Table 3:** Learning Points MALT: mucosa-associated lymphoid tissue

Learning Points
Gastric MALT lymphoma can occur in patients who are H. pylori negative.
Eradication therapy is a potential initial therapy in H. pylori negative patients. It is less aggressive, cheaper, and has less side effects and complication risks compared to radiation, monoclonal antibody therapy, and chemotherapy.
Although extremely rare, patients with gastric MALT lymphoma can initially present with hemodynamically unstable gastrointestinal bleeding.

## Conclusions

Gastric MALT lymphoma is associated with H. pylori gastritis; however it can also occur in patients who are H. pylori negative. In cases of H. pylori negative gastric MALT lymphoma, studies suggest that eradication therapy may be considered as an initial treatment modality. Studies have shown that eradication therapy has successfully cured patients of localized disease despite being H. pylori negative. It is important to note that eradication therapy is not currently considered the standard of care and randomized prospective studies are needed to further explore the efficacy of this treatment option in patients with gastric MALT lymphoma who do not have H. pylori. Eradication therapy is cheaper, less invasive, and has less side effects compared to radiotherapy, chemotherapy, and monoclonal antibodies. Therefore, it is worth considering eradication therapy as a first choice of treatment in these patients. Although extremely rare, patients with gastric MALT lymphoma can initially present with hemodynamically unstable gastrointestinal bleeding. 
